# Exploiting the microbiota of organic and inorganic acid-treated raw poultry products to improve shelf-life

**DOI:** 10.3389/fmicb.2024.1348159

**Published:** 2024-02-27

**Authors:** Dana K. Dittoe, Kristina M. Feye, Christina Ovall, Hayley A. Thompson, Steven C. Ricke

**Affiliations:** ^1^Animal Science, University of Wyoming, Laramie, WY, United States; ^2^Cell and Molecular Biology Program, University of Arkansas, Fayetteville, AR, United States; ^3^Jones-Hamilton Co., Walbridge, OH, United States; ^4^Center for Food Safety, Department of Food Science, University of Arkansas, Fayetteville, AR, United States; ^5^Meat Science and Animal Biologics Discovery Program, Animal and Dairy Sciences, University of Wisconsin, Madison, WI, United States

**Keywords:** shelf life, microbiome, poultry, peracetic acid, sodium bisulfate

## Abstract

**Introduction:**

Targeted amplicon sequencing of the 16S rRNA delineates the complex microbial interactions that occur during food spoilage, providing a tool to intensively screen microbiota response to antimicrobial processing aids and interventions. The current research determines the microbiota and spoilage indicator (total aerobes and lactic acid bacteria; LAB) response to inorganic and organic antimicrobial intervention use on the shelf-life of fresh, never-frozen, skin-on, bone-in chicken wings.

**Methods:**

Wings (*n*=200) were sourced from local processor and either not treated (NT) or treated with 15-s dips of tap water (TW), organic (peracetic acid; PAA), inorganic acids (sodium bisulfate; SBS), and their combination (SBS + PAA). Wings were stored (4°C) and rinsed in neutralizing Buffered Peptone Water (BPW) for 1 min on d 0, 7, 14, and 21 post-treatment. Spoilage indicators, aerobic mesophiles and LAB, were quantified from rinsates. Genomic DNA of d 14 and 21 rinsates were extracted, and V4 of 16S rRNA gene was sequenced. Sequences were analyzed using QIIME2.2019.7. APC and LAB counts were reported as Log_10_ CFU/g of chicken and analyzed in R Studio as a General Linear Model using ANOVA. Pairwise differences were determined using Tukey’s HSD (P£0.05).

**Results:**

Spoilage was indicated for all products by day 21 according to APC counts (>7 Log_10_ CFU/g); however, wings treated with SBS and SBS + PAA demonstrated a 7-day extended shelf-life compared to those treated with NT, TW, or PAA. The interaction of treatment and time impacted the microbial diversity and composition (*p* < 0.05), with those treated with SBS having a lower richness and evenness compared to those treated with the controls (NT and TW; *p* < 0.05, Q < 0.05). On d 14, those treated with SBS and SBS + PAA had lower relative abundance of typical spoilage population while having a greater relative abundance of *Bacillus* spp. (~70 and 50% of population; ANCOM *p* < 0.05). By d 21, the *Bacillus* spp. populations decreased below 10% of the population among those treated with SBS and SBS + PAA.

**Discussion:**

Therefore, there are differential effects on the microbial community depending on the chemical intervention used with organic and inorganic acids, impacting the microbial ecology differently.

## 1 Introduction

Spoilage is a significant cost and concern for the poultry industry (Manning et al., 2007). In congruence with pathogen mitigation strategies, the poultry industry employs multiple technologies to extend the shelf life of poultry products (Souza et al., 2018; Baltic et al., 2019; Karaca et al., 2023). Antimicrobial agents, such as organic and inorganic acids, chlorine, and bromide-based chemicals, are used throughout poultry processing as sprays or immersion dips on whole birds or parts (Dittoe et al., 2019a,b; Feye et al., 2019; Micciche et al., 2019). However, due to the multiple selective pressures created by differing modes of action associated with each varying antimicrobial treatment, the frequent application of these chemical interventions used within a multi-hurdle approach alters or modifies the microbial ecology of poultry products (Rahman, 2015). In addition to selective pressures endured at the time of application, residues resulting from the extensive half-life of the processing antimicrobials on meat exist (Walsh et al., 2018).

With current methodologies employed by the poultry industry and the United States Department of Agriculture – Food Safety Inspection Service (USDA – FSIS), the impact these antimicrobial extension efforts have on the microbial ecology of the raw meat product is not a routine measurement of efficacy (Ricke et al., 2018). Traditionally, these routine measurements of shelf-life extension efforts include microbial plating of indicator organisms such as total mesophilic aerobic bacteria (>7 Log_10_ CFU/ml of poultry carcass rinsates) and lactic acid bacteria (LAB; > 6.8 Log_10_ CFU/ml of carcass rinsates; Balamatsia et al., 2007). However, these measurements do not provide insight into the early and late onset changes that impact the microbiota due to the chemical interventions utilized during or after processing. Due to the chemical composition and mode of action with these interventions, certain microorganisms associated with spoilage may be increased but not necessarily detected in mesophilic aerobic bacteria and LAB counts. For example, specific acid-tolerant spoilage populations, acetic acid bacteria, and acid-tolerant spore-forming spoilage bacteria may remain after treatment with acidic compounds, such as peracetic acid (Tran et al., 2020; Sun et al., 2021); however, these population differences will not be demonstrated in the indicator microorganisms, mesophilic aerobic bacteria, and LAB. Therefore, elucidating the microbiota composition of treated products over time and determining the microbiota’s stability may provide a more accurate understanding of how these chemical interventions impact shelf-life extension.

Recently, the use of sodium bisulfate (SBS) has been evaluated in previous studies for its ability to reduce experimentally inoculated foodborne pathogens on processed poultry parts (Dittoe et al., 2019a). The use of SBS has also been demonstrated to reduce premature browning and extend the shelf-life of apples (Fan et al., 2009; Kim et al., 2018). While it is an efficacious antimicrobial, the effect it has on the shelf-life of poultry as compared with the industry standard is not defined. Therefore, the objective of the current study was to determine the microbial quality of poultry wings over an extended period (21 days) after a short duration (15 s) treatment of either peroxyacetic acid (PAA), an organic acid and peroxide, or sodium bisulfate (SBS), an inorganic acid, as defined by traditional microbial plating (LAB and APC) and microbiome sequencing. Traditional microbial plating of spoilage bacteria (LAB) coupled with total microbial load (APC) may indicate microbial shifts that proceed to spoilage that correspond with the underlying microbiota dynamics. Ultimately, this study hopes to preliminarily evaluate the antimicrobial effects of SBS and PAA and their combination on the microbial ecology of poultry products and how these antimicrobials may impact the corresponding shelf-life using traditional microbiological testing methods.

## 2 Materials and methods

### 2.1 Treatment preparation and application

A total of 200 wings (5 treatments, 4 time points, and 10 replicates) were obtained from a local chicken processor in northwest Arkansas. Wings were procured no longer than 24 h before the onset of the study. The bone-in, skin-on-whole chicken wings had a mean weight of 114.74 g with a standard error of 5.16 g.

The tap water used in the current experiment was not treated (un-sterilized) and was obtained from the Center for Food Safety at the University of Arkansas, which is supplied by the Beaver Water District (Lowell, AR, United States) that distributes water across northwest Arkansas. The water was reported to have less than 1 ppm of chlorine, fluoride, and nitrates and < 1 ppm of lead and copper (City of Fayetteville Arkansas, 2020). Therefore, the quality of the water should not have interfered with the chemistry of antimicrobial treatments. However, the water was not sterilized, which could have allowed the introduction of microorganisms. The tap water was not altered or sterilized in order to mimic industry settings.

The five experimental antimicrobial treatments were created by combining 15 L of tap water with either 3% sodium bisulfate (w/v; SBS, Jones-Hamilton Co., Walbridge, OH, United States), 500 ppm of peroxyacetic acid (Spectrum® 22, PeroxyChem, Philadelphia, PA, United States), or the combination of them to create the following treatments: a no treatment control (NT), Tap Water alone (TW), TW with the inclusion of 3% (w/v) SBS (SBS), TW with the addition of 500 ppm of PAA (PAA), and the combination of TW, SBS, and PAA (SBS + PAA). The treatment TW was included to demonstrate the rinsing effect of TW without the addition of antimicrobial treatments. A SympHony pH meter and probe (VWR International, Radnor, PA, United States) were used to determine the pH of the solutions (TW: 8.52 pH; SBS: 1.20 pH; PAA: 3.62 pH; SBS + PAA: 1.20 pH).

Subsequently, 350 mL of the treatments was aliquoted to sterile whirlpak bags for treatment application. The weight of the whole chicken wings was recorded and then independently dipped into sterile collection bags for 15 s (VWR International, Radnor, PA, United States) containing the five previously described treatments. Treatments were applied at ambient room temperature (20–22°C).

### 2.2 Microbial analysis: total mesophilic aerobes and lactic acid bacteria enumeration

After the wings were treated in antimicrobial dips for 15 s, they were aseptically shaken (10–15 s) within the sterile collection bag to allow excess treatments to drip off prior to storage in new sterile collection bags. On d 0, the treated wings were allowed to rest for 2 min in the new collection bags prior to microbial analysis (Dittoe et al., 2019a). Immediately following the rest period, the wings were either evaluated immediately on d 0 or maintained at 4°C until d 7, 14, or 21 and analyzed for a total load of mesophilic aerobic bacteria and LAB per gram of wing. At each time point after treatment, d 0, 7, 14, and 21, the wings were rinsed with 150 mL of sterile neutralizing Buffered Peptone Water (nBPW; 20.0 g of buffered peptone, 7 g of refined soy lecithin or equivalent, 1.0 g of sodium thiosulfate, 12.5 g of sodium bicarbonate, per 1 L of DI water; 20–22°C; USDA FSIS, 2019). Wings were manually agitated for 1 min, with the resulting rinsates being collected for downstream analysis while the wings were discarded. Rinsates were aliquoted (1.0 mL) in two 1.5 mL microcentrifuge tubes and either maintained at 4°C for mesophilic aerobes and LAB enumeration or at −80°C for 16S rRNA gene sequencing.

Whole chicken wing rinsates were subsequently serially diluted to 10^−7^ (1:10 dilution factor) in 96-well plates (25 μL of rinsate in 225 μL of 1 × Phosphate Buffered Saline, PBS). After dilution, 10 μL of each dilution was dot plated onto Tryptic Soy Agar (TSA, BD Difco™, Franklin Lakes, NJ, United States) and De Man, Rogosa, and Sharpe agar (MRS, BD Difco™, Franklin Lakes, NJ, United States) in duplicate (Jett et al., 1997; Herigstad et al., 2001; Naghili et al., 2013). The plates were dried, inverted, and incubated aerobically for 24 h at 37°C or 48 h anaerobically at 37°C, respectively. Plated dilutions with CFU counts between 6 and 60 were enumerated.

The CFU of bacteria per gram of treated whole chicken wings on days 0, 7, 14, and 21 was calculated using the equation described by Dittoe et al. (2019a):


$$\frac{\left(\frac{Numberofcolonies}{volumeplated}\right)*\mathrm{Dilution Factor}}{\frac{\mathrm{Wing Weight}\;\left(g\right)}{\mathrm{Original Homogenate}\;\left(\mathrm{mL}\right)}}=\mathrm{CFU}/\mathrm{gram of Wing}$$

### 2.3 Microbiome analysis: 16S rRNA gene sequencing

On 14 and 21 d post-treatment, rinsates were aliquoted to 1.5 mL microcentrifuge tubes and stored at −80°C until DNA extraction could occur. Following the Gram-Negative Bacteria protocol of the QIAGEN DNeasy Blood and Tissue Kit (QIAGEN, Hilden, Germany), a subset of aliquoted rinsates (*n* = 5) was thawed, centrifuged for 10 min at 5,000 × g, and decanted to remove fat and buffer from the pelleted microorganisms. The pellet was resuspended in 180 μL of ATL buffer. Following, the DNA was extracted using the standard protocol with DNA eluted in 30 L of Buffer AE. The DNA purity and quality were assessed using a Nanodrop™ 1,000 spectrophotometer (Thermo Scientific, Waltham, MA, United States). Subsequently, DNA was diluted to 10 ng/μl in Buffer AE.

Libraries were constructed according to Kozich et al. (2013), where dual-index paired-end primers targeting V34, V4, and V45 regions of the bacterial 16S rRNA gene and a high-fidelity polymerase (AccuPrime Pfx SuperMix, Invitrogen, Carlsbad, CA, United States) were used. Amplicons in uniform concentrations (18 μL) were normalized using SequalPrep Normalization Kit (Invitrogen, Carlsbad, CA, United States) and quantified using a Qubit fluorometer and kit (Invitrogen, Carlsbad, CA, United States). Following, normalized amplicons were pooled together in equimolar concentrations into a 1.5 mL microcentrifuge tube and quantified again using the Qubit system. The final concentration of the library was verified through the use of quantitative PCR (qPCR) with the KAPA library quantification kit for Illumina platforms (KAPA Biosystems, Wilmington, MA, United States) using SYBR green technology. Amplicon size was determined using a bioanalyzer (Agilent, Santa Clara, CA, United States).

The library was diluted to 20 μM in HT1 buffer (Illumina, San Diego, California, United States) and denatured with NaOH (0.2 N). The diluted library was mixed with 10% PhiX (Illumina, San Diego, California, United States) and loaded into a MiSeq v2 500 cartridge (Illumina, San Diego, California, United States). The cartridge was then loaded into an Illumina MiSeq (Illumina, San Diego, California, United States) sequencer with subsequent sequences uploaded to BaseSpace (Illumina, San Diego, California, United States), NCBI Sequence Read Archive (PRJNA847567), and GitHub.^1^

### 2.4 Statistical analysis

Before the onset of the experiment, a treatment and a time point were randomly assigned to each wing. Only the CFU of total mesophilic aerobes and LAB between 6 and 60 were recorded, with those below the limit of detection (6 × 10^2^ CFU/mL) being recorded as 6 × 10^2^ CFU/mL. Total mesophilic aerobes and LAB (CFU/ml) were calculated as CFU per gram of wing and, subsequently, Log_10_ transformed. The data were assessed for normality, satisfying the assumptions of a linear model in R Studio (R version 4.3.2; R Core Team, 2023). As the same wing was not continuously sampled throughout time, the microbial count data were analyzed as a randomized complete block design with day designated as the block. The main effects of day and treatment and the interaction were analyzed using a general linear model. Pairwise differences were determined using Tukey’s protected HSD at a *p* ≤ 0.05 level of significance. Figures of the microbiological count data were generated in R Studio with a linear line at 7 Log CFU/g added to demonstrate the onset of spoilage (Balamatsia et al., 2007).

### 2.5 Bioinformatics

Demultiplexed amplicon sequences were downloaded from BaseSpace and imported into QIIME2 2019.7 (Bolyen et al., 2019) using Casava 1.8 paired-end demultiplexed format (via qiime tools import), where microbiome informatics were performed on rinsates collected on d 14 and 21. Quality filtering and denoising of demultiplexed sequences were performed using DADA2 (Callahan et al., 2016). The amplicon sequence variants (ASVs) were aligned with SEPP (via q2-fragment insertion) (Matsen et al., 2010; Eddy, 2011; Janssen et al., 2018). Using fasttreee2 (via q2-phylogeny), a rooted phylogenetic tree was generated (Price et al., 2010). The ASVs were putatively identified using SILVA (Quast et al., 2013; Yilmaz et al., 2014; Glöckner et al., 2017) with the sk-learn Bayesian algorithm, which accounts for the error rate associated with sequencing, alignment, and upstream plug-ins (via q2-feature-classifier) (Bokulich et al., 2018a). Populations were rarified to the average sequencing depth (100,000) and visualized for saturation purely to select the point at which alpha and beta diversity analysis could be performed while maintaining all samples (via q2-diversity). As such, the core alpha and beta metrics were performed at a sequencing depth of 200, which retained 10,000 (0.19%) features in all 50 (100.00%) samples at the specified sampling depth (Supplementary Figure S1). Alpha diversity was analyzed using the traditional pipeline, with the main effects and interactions identified using ANOVA (via q2-longitudinal) (Bokulich et al., 2018b) and pairwise comparisons using Kruskal–Wallis for Shannon’s Diversity Index and (Shannon, 1948; Pielou, 1966). Beta diversity metrics, Weighted and Unweighted Unifrac, were also analyzed using the traditional pipeline with ANOSIM (Lozupone and Knight, 2005; Lozupone et al., 2007; Oksanen et al., 2018), with the main effects and interactions identified using ADONIS (Anderson, 2001). Volatility plots were calculated via q2-longitudinal. Compositional differences were evaluated using ANCOM (via q2-composition) (Mandal et al., 2015). Final ANCOM tables were visualized in Microsoft Excel (Microsoft, Redmond, WA, United States).

## 3 Results

### 3.1 Peracetic acid and SBS impact on shelf-life

**Figure 1:**
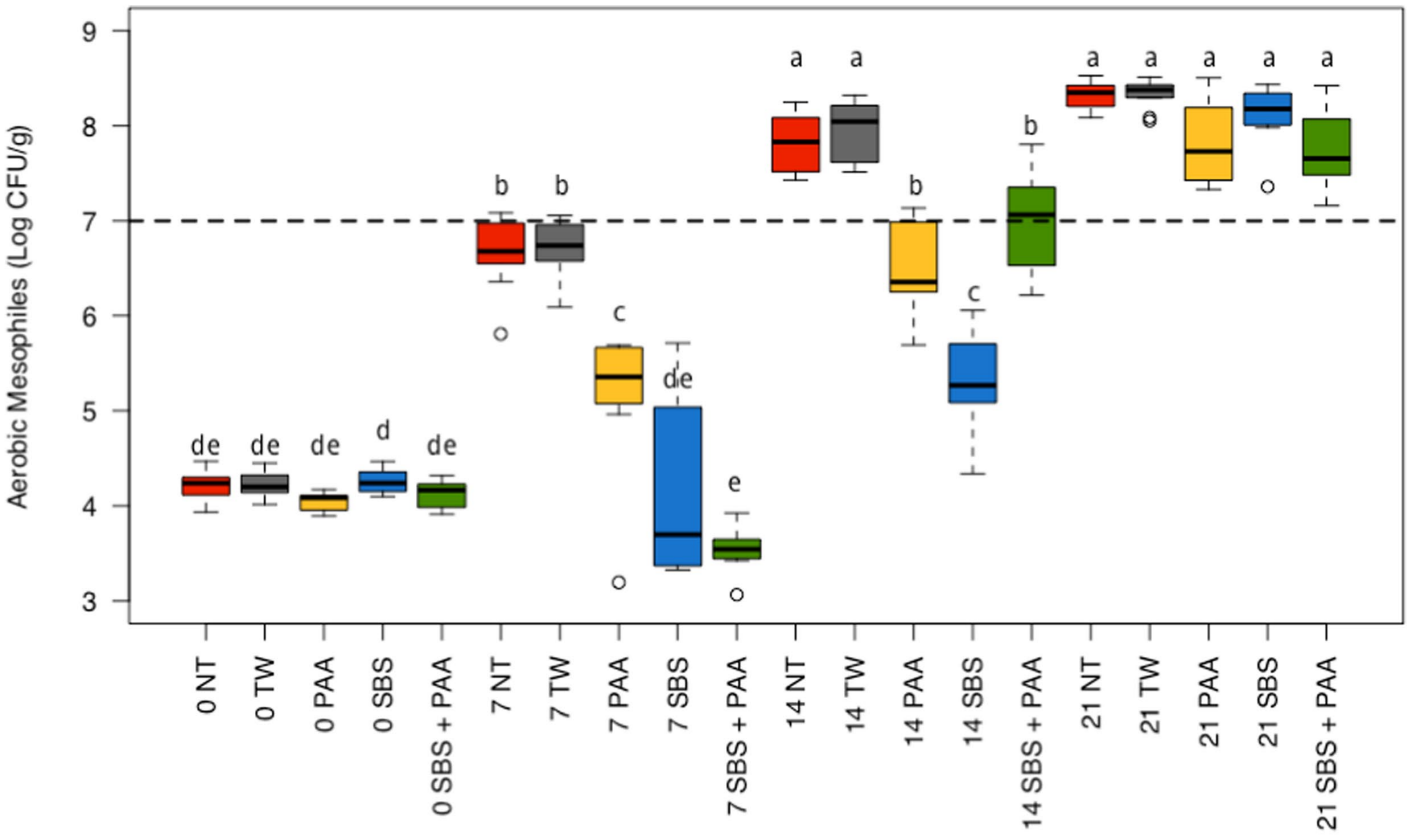
The interaction between treatment and day on the total load of mesophilic aerobes on chicken wings treated with organic and inorganic acids over a 21-d period (*p* < 0.05). The distribution of the data was captured using boxplots with outliers. Treatments were applied as 15-s dips into either with no treatment (NT), tap water (TW), TW + 500 ppm peracetic acid (PAA), TW + 3% Sodium Bisulfate (SBS), and the combination of TW + SBS + PAA (SBS + PAA) on d 0. The dashed line represents the theoretical onset of spoilage, 7 Log_10_ CFU/g. Those treated as NT, TW, PAA, SBS, and SBS + PAA are represented as red, grey, yellow, blue, and green boxplots, respectively. Those with different connecting letters (a-e) are considered significantly different (*p* < 0.05). Exact mean and standard error of the mean Log_10_ CFU/g values are presented in Supplementary Table S1.

There was an interaction between treatment and time on the aerobic mesophiles recovered from non-treated and treated chicken wings (*p* < 0.05; Figure 1; Supplementary Table S1). At day 0, there were no differences between treatments (*p* > 0.05). On d 7, chicken wings treated with PAA, SBS, and SBS + PAA had significantly lower concentrations of aerobic mesophiles than those treated as NT or TW (*p* < 0.05). In addition, those treated with PAA had higher concentrations of aerobic mesophiles on d 7 than those treated with SBS and SBS + PAA (*p* < 0.05). On d 14, there were fewer aerobic mesophiles recovered from those treated with PAA, SBS, and SBS + PAA than those treated with the controls, NT, and TW (*p* < 0.05). On d 21, there were no differences in the concentration of recovered aerobic mesophiles (*p* > 0.05).

Those treated with NT and TW demonstrated an increase in aerobic mesophiles from d 0 to d 7 (~ + 2.50 Log_10_ CFU/g) and d 7 to d 14 (~ + 1.10 Log_10_ CFU/g) and did not differ from d 14 to d 21 (*p* < 0.05; Figure 1). Those treated with PAA had an increase in recovered aerobic mesophiles at each time-point until d 21 (~ + 1 Log_10_ CFU/g per 7 d). Chicken wings treated with SBS and SBS + PAA did not have an increase in aerobic mesophiles until d 7 and then increased until d 21 (*p* < 0.05).

**Figure 2:**
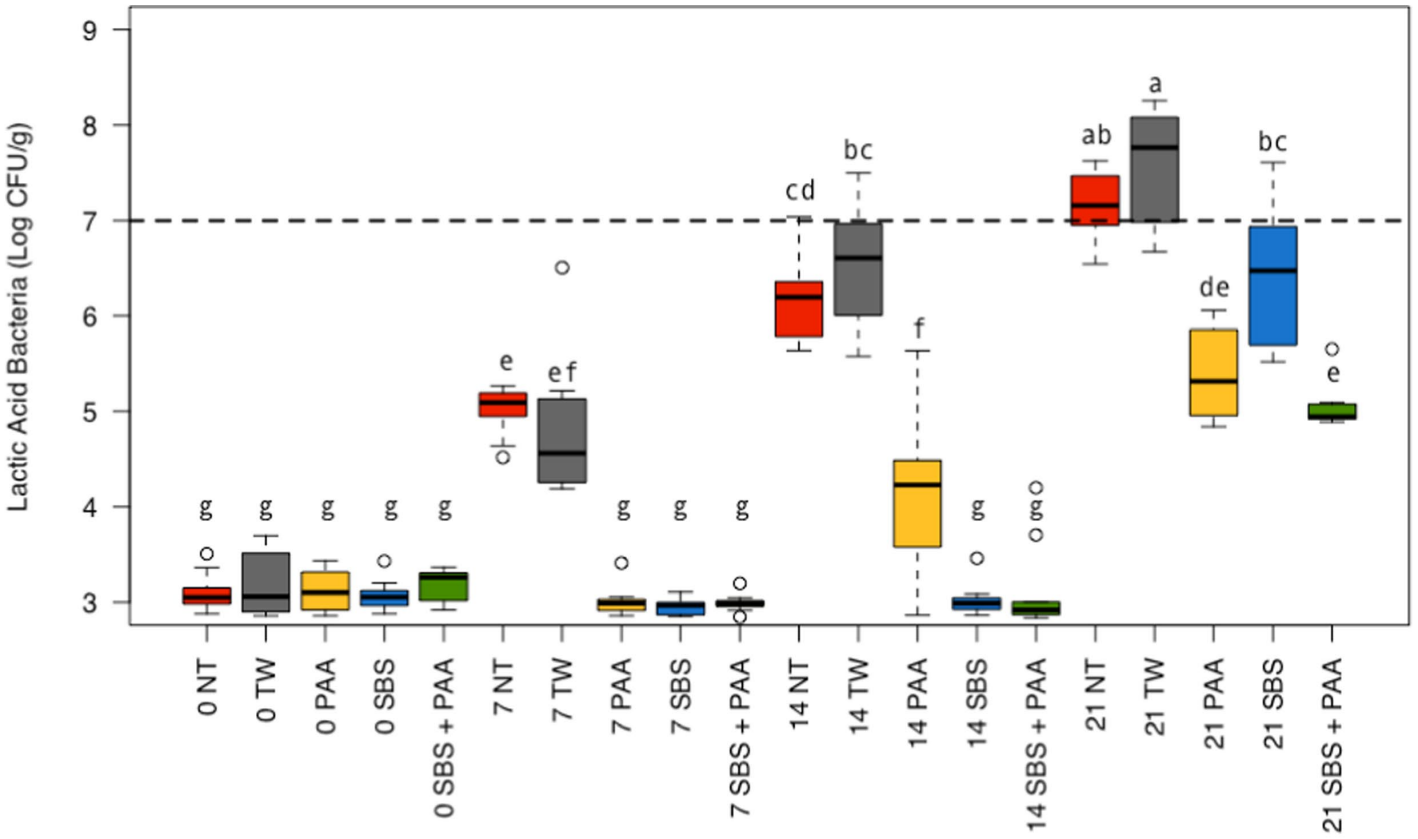
The interaction between treatment and day on the total load of lactic acid bacteria of chicken wings treated with organic and inorganic acids over a 21-d period (*p* < 0.05). Treatments were applied as 15-s dips into either with no treatment (NT), tap water (TW), TW + 500 ppm peracetic acid (PAA), TW + 3% Sodium Bisulfate (SBS), and the combination of TW + SBS + PAA (SBS + PAA) on d 0. The distribution of the data was captured using boxplots with outliers with the dashed line representing the theoretical onset of spoilage, 7 Log_10_ CFU/g. Those treated as NT, TW, PAA, SBS, and SBS + PAA are represented as red, grey, yellow, blue, and green boxplots, respectively. Those with different connecting letters (a-e) are considered significantly different (*p* < 0.05). Exact mean and standard error of the mean Log_10_ CFU/g values are presented in Supplementary Table S1.

There was also an interaction between treatment and time on the LAB recovered from chicken wings (*p* < 0.05; Figure 2; Supplementary Table S1). There was no difference between those not treated, NT and TW, and those treated, SBS, PAA, and SBS + PAA, on d 0 (*p* > 0.05). On d 7, those treated, SBS, PAA, and SBS + PAA, had less recoverable total LAB than those not treated, NT and TW (*p* < 0.05). On d 14, those treated also had less recoverable LAB than those treated as controls. On d 14, those treated with SBS and SBS + PAA had lower recoverable LAB than those treated with PAA (*p* < 0.05). On d 21, only those treated with PAA or SBS + PAA had lower LAB recovered than both of the controls; however, those treated with SBS did have lower LAB than those treated with TW (*p* < 0.05). Although wings treated with PAA and SBS + PAA did not have different LAB levels (Log_10_ CFU/g) on d 21 (*p* > 0.05), those treated with SBS + PAA had the lowest LAB load on d 21.

Over time, all the levels of LAB increased among all treated wings; however, those treated with SBS and SBS + PAA did not increase until d 21, with recovered LAB being consistent from d 0 to d 14 (Figure 2). Those treated with PAA did not have an increase in LAB from d 0 to d 7, but from d 7 to d 14 and d 14 to d 21, there was an increase in LAB (~ + 1 Log_10_ CFU/g). The controls increased at each time-point (*p* < 0.05).

### 3.2 Treatment, but not time, impacted alpha diversity

**Figure 3:**
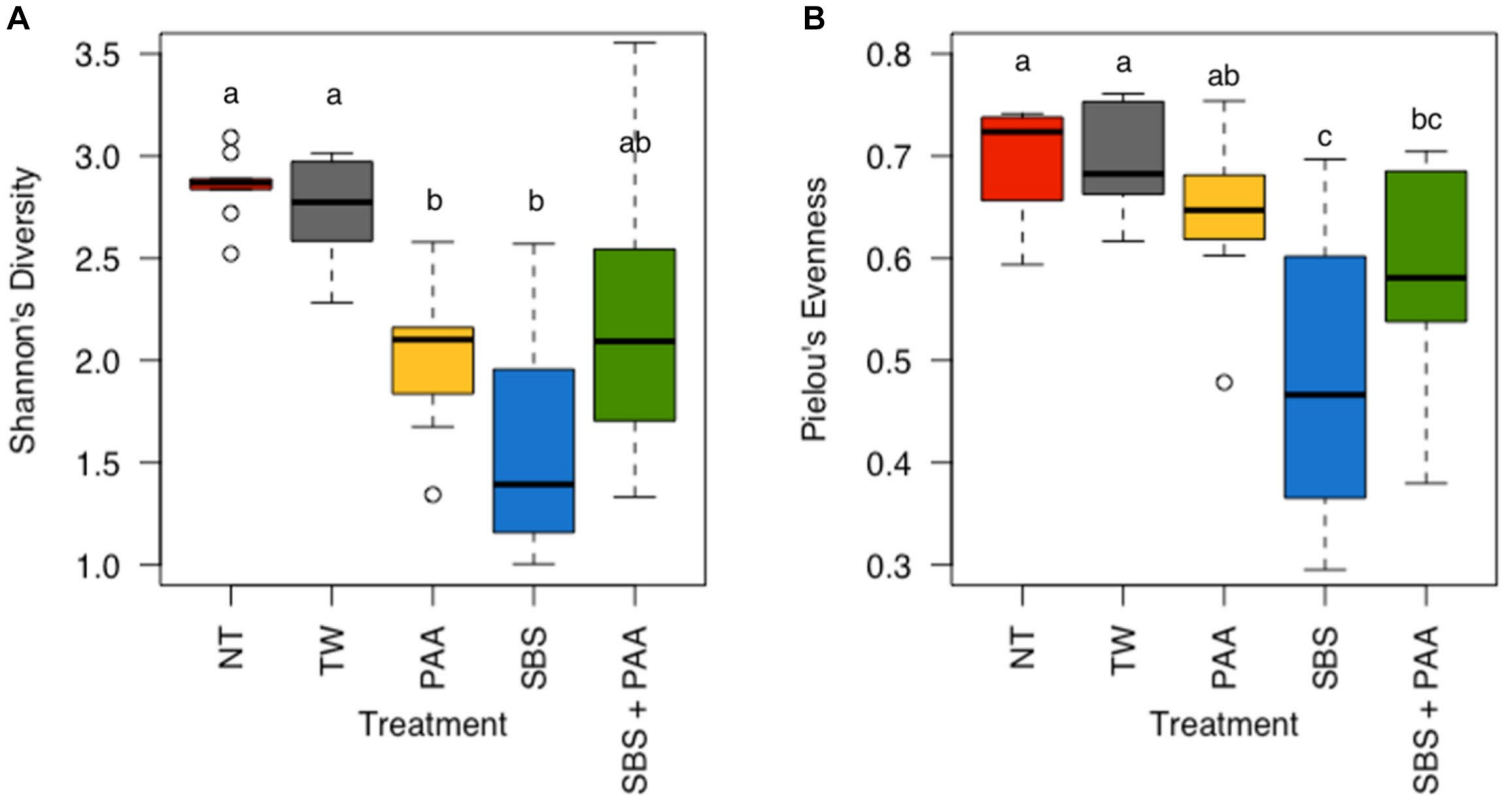
The richness and evenness of the microbiota of the rinsates of bone-in, skin-on chicken wings treated with no treatment (NT), tap water (TW), TW + 500 ppm peracetic acid (PAA), TW + 3% Sodium Bisulfate (SBS), and the combination of TW + SBS + PAA (SBS + PAA). There was a main effect of treatment on both Shannon’s Entropy **(A)** and Pielou’s Evenness **(B)** of the microbiota recovered from the chicken wing poultry rinsates (*N* = 50, *n* = 10, *k* = 5, *p* < 0.05). Those treated as NT, TW, PAA, SBS, and SBS + PAA are represented as red, grey, yellow, blue, and green boxplots, respectively. Those with different connecting letters (a-c) are considered significantly different (Q < 0.05; Supplementary Tables S2, S3).

There was neither any interaction between treatment and day nor the main effect of day on the richness (Shannon’s Entropy) and evenness (Pielou’s Evenness) of the rinsates of bone-in, skin-on chicken wings (Supplementary Table S2; *p* > 0.05). However, there was a treatment effect on the richness and evenness of the wings (Supplementary Table S2; *p* < 0.001 and *p* = 0.02). Those treated with PAA and SBS (2.02 ± 0.11 and 1.60 ± 0.18 Shannon’s Entropy) had a lower richness than those not treated (2.85 ± 0.05 and 2.74 ± 0.08 Shannon’s Entropy) (*p* < 0.05, Q < 0.05), with those treated with SBS + PAA (2.24 ± 0.23 Shannon’s Entropy) not being different than those untreated or treated (Figure 3A; Supplementary Table S3). Those treated with SBS and SBS + PAA (0.47 ± 0.04 and 0.58 ± 0.03 Pielou’s Evenness) had a lower evenness than those untreated (0.70 ± 0.02 and 0.69 ± 0.02 Pielou’s Evenness). Those treated with SBS + PAA did not differ in evenness from those treated with PAA (0.64 ± 0.02 Pielou’s Evenness; (*p* > 0.05; Q > 0.05) Figure 3B).

### 3.3 Beta volatility over time

**Figure 4:**
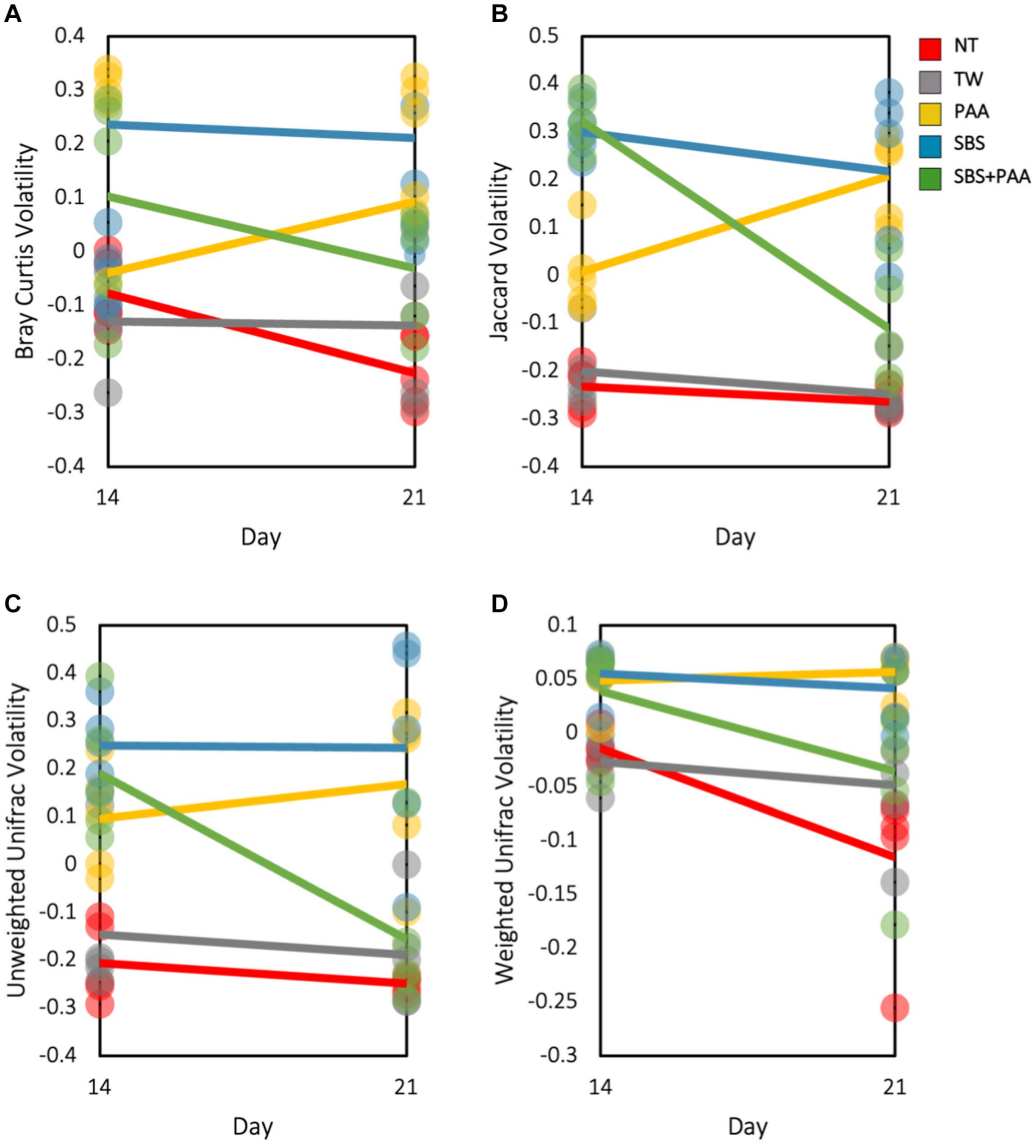
The beta volatility of the microbiota of the rinsates of bone-in, skin-on chicken wings treated with no treatment (NT), tap water (TW), TW + 500 ppm peracetic acid (PAA), TW + 3% Sodium Bisulfate (SBS), and the combination of TW + SBS + PAA (SBS + PAA) on d 14 and 21 post-treatment. An interaction between treatment × day on Bray–Curtis **(A)**, Jaccard Dissimilarity Index **(B)**, Unweighted Unifrac **(C)**, and Weighted Unifrac **(D)** of the microbiota recovered from the chicken wing poultry rinsates was observed (*N* = 50, *n* = 10, *k* = 5; *p* = 0.045, 0.004, 0.025, and 0.154).

There was an interaction between treatment and day on the Bray–Curtis, Jaccard Dissimilarity, and Unweighted Unifrac (*p* < 0.05; Supplementary Table S4). Treatment and time had a main effect on Weighted Unifrac (*p* > 0.05). To explore this interaction, the volatility of the beta diversity metrics was determined on d 14 and 21 (Figure 4). Those treated with SBS had a stable volatility over time with a higher Bray–Curtis, Jaccard, and Unweighted Unifrac volatility than wings treated with NT, TW, PAA, and SBS + PAA. Those treated with PAA increased in volatility in all beta diversity metrics, whereas those treated with the combination of SBS + PAA increased in Bray–Curtis, Jaccard Dissimilarity, and Unweighted Unifrac volatility over time (Figures 4A–C). Wings designated as NT had a decrease in Bray–Curtis and Weighted Unifrac volatility from d 14 to d 21 (Figures 4A,C). Those designated as TW did not change in beta diversity volatility over time.

**Figure 5:**
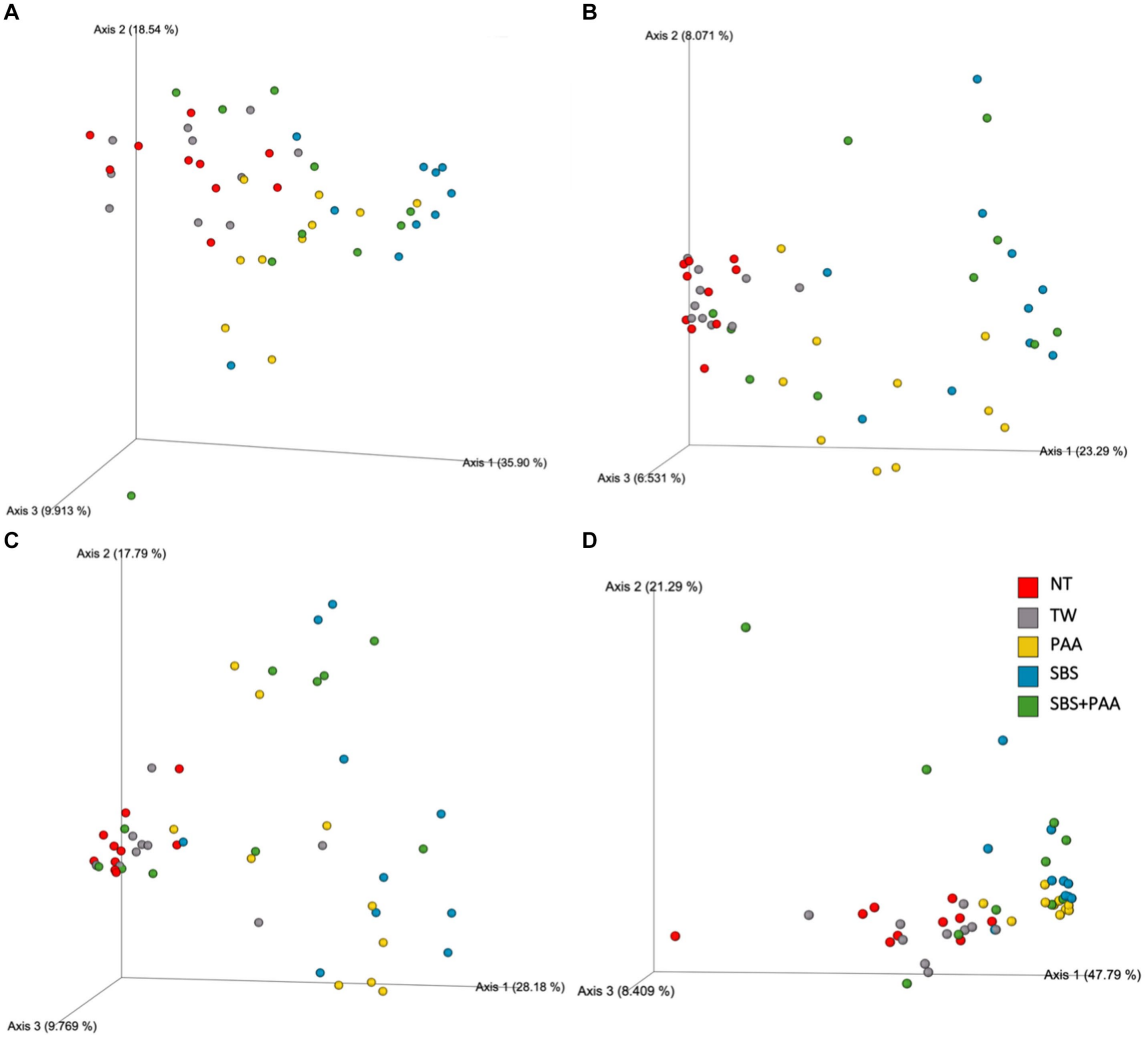
The beta diversity of the microbiota of the rinsates of bone-in, skin-on chicken wings treated with no treatment (NT), tap water (TW), TW + 500 ppm peracetic acid (PAA), TW + 3% Sodium Bisulfate (SBS), and the combination of TW + SBS + PAA (SBS + PAA) as represented in a three-dimensional plot, PcoA. A main effect of treatment on Bray–Curtis **(A)**, Jaccard Dissimilarity Index **(B)**, Unweighted Unifrac **(C)**, and Weighted Unifrac **(D)** of the microbiota recovered from the chicken wing poultry rinsates was observed (*N* = 50, *n* = 10, *k* = 5, *p* < 0.05). Pairwise differences are represented in Supplementary Table S5.

With treatment being a driving factor on the beta diversity of the untreated and treated bone-in, skin-in chicken wings, 3-dimensional plots (PCoA) were utilized to depict the effect of treatments on the beta diversity of the wings with pairwise differences being determined by ANOSIM (Figure 5; Supplementary Table S5). In all beta diversity metrics, those designated as the controls, NT, and TW did differ and were different than all wings treated with PAA, SBS, and SBS + PAA (*p* > 0.05; Q > 0.05; Figure 5; Supplementary Table S5). The Bray–Curtis and Jaccard Dissimilarity of those treated with PAA and SBS were different (*p* < 0.05, Q < 0.05; Figures 5A,B). There was no difference between the Unweighted and Weighted Unifrac of those treated with PAA and SBS (*p* > 0.05, Q > 0.05; Figures 5C,D). The Bray–Curtis and Weighted Unifrac of those treated with SBS + PAA were different than PAA, but only the Bray–Curtis of those treated with SBS + PAA was different than SBS (*p* < 0.05, Q < 0.05; Figures 5A,D; Supplementary Table S5).

### 3.4 Compositional changes by time

**Figure 6:**
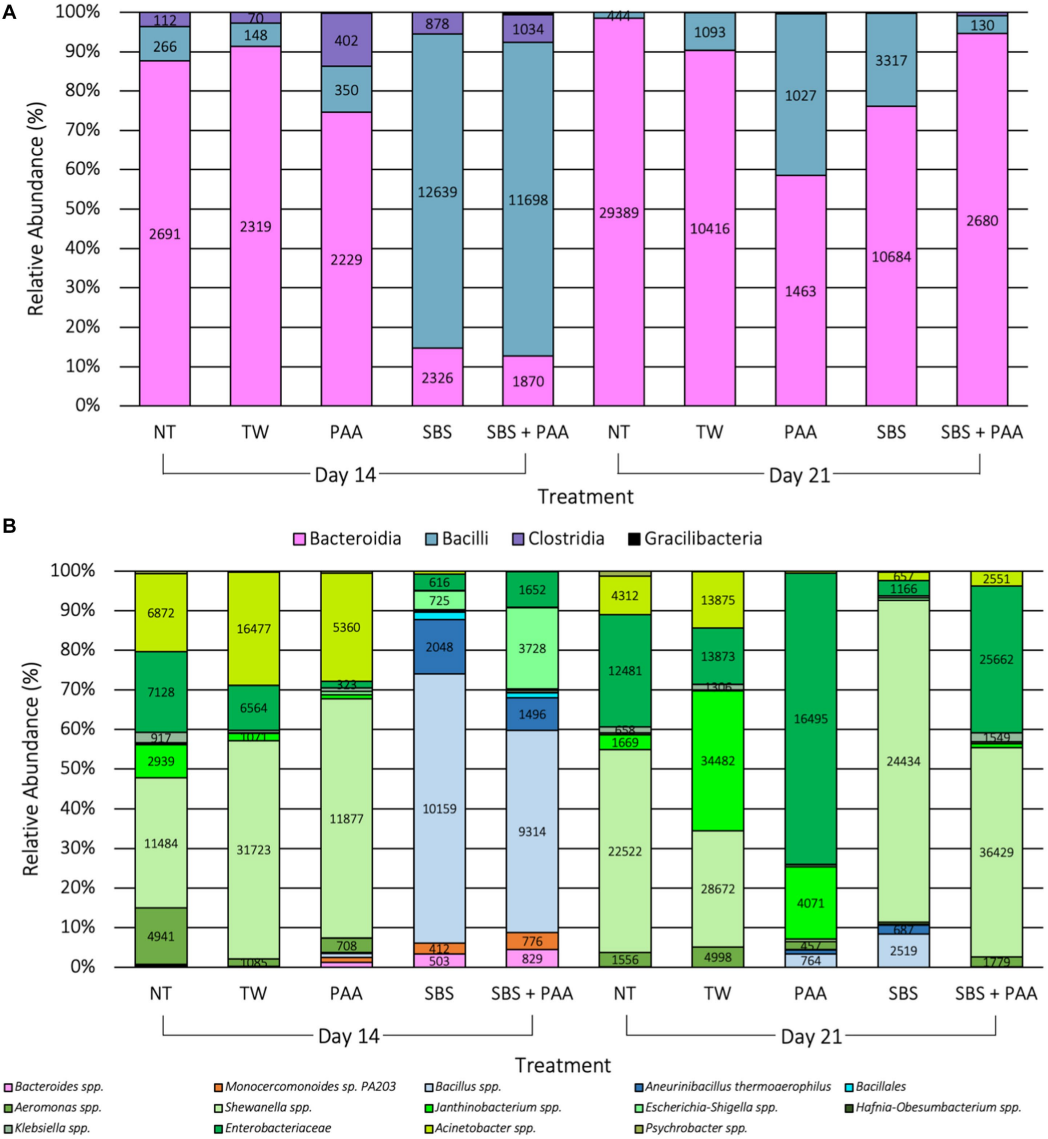
Significant taxa at the class **(A)** and species **(B)** levels among the microbiota of the rinsates of bone-in, skin-on chicken wings treated with no treatment (NT), tap water (TW), TW + 500 ppm peracetic acid (PAA), TW + 3% Sodium Bisulfate (SBS), and the combination of TW + SBS + PAA (SBS + PAA) on d 14 and 21 post-treatment (*p* < 0.05, W > 59). Taxa belonging to Bacteroidetes are pink, Bacilli are blue, Clostridia are purple, Alphaproteobacteria are yellow, Verrucomicrobia are beige, and Gammaproteobacteria are green.

There were 54 unique taxa identified at the class level, and 479 unique taxa were identified at the species level. There were 4 differentially abundant taxa (10 < W > 8) at the class level and 14 differentially abundant taxa (70 < W > 59) at the species level on d 14 and d21 due to treatment application when using ANCOM, the analysis of compositions of microbiomes (*p* < 0.05; Figure 6). At the class level, Bacilli, Clostridia, Bacteroidia, and Gracilibacteria were differentially abundant than 10, 10, 9, and 8 other taxa at the class level (*p* < 0.05; Figure 6A). At the species level, *Aneurinibacillus thermoaerophilus, Monocercomonoides* sp. PA203, *Bacillus*, *Bacillales*, *Acinetobacter*, *Shewanella*, *Escherichia-Shigella*, *Janthinobacterium*, *Klebsiella*, *Psychrobacter*, *Aeromonas*, *Hafnia-Obesumbacterium*, *Bacteroides*, and *Enterobacteriaceae* were differentially abundant than, 70, 69, 68, 68, 68, 67, 66, 65, 62, 62, 61, 60, 60, and 59 other taxa at the species level (*p* < 0.05; Figure 6B). Those treated with SBS and SBS + PAA had a higher relative abundance of *Bacilli* at the class level on d 14 than those not treated or those treated with PAA, which translated to higher levels of *Bacillales* and *Bacillus* spp. at the species level (Figure 6). By d 21, though there were still populations of bacilli, the populations of Bacteroidia at the class level and populations of *Shewanella* spp. at the species level were highly prevalent among the significantly differentially abundant taxa (Figure 6).

## 4 Discussion

### 4.1 Peracetic acid and SBS extend shelf-life and reduce microbial load over time

The noticeable spoilage (off odors, discoloration, and slime production) of poultry typically occurs when the total mesophilic aerobic bacteria and LAB populations reach approximately 7 Log_10_ CFU/g of product (Mead, 2007; Nychas et al., 2008). Commonly, the measurement of total aerobic bacteria and LAB (< 7 Log_10_ CFU/g) to determine the shelf-life of poultry is utilized with the application of this metric as the preliminary step to identify spoilage. In the current study, culture-based quantification of these indicator organisms was the first step in determining the shelf-life of bone-in, skin-on chicken wings with the end of shelf-life microbiota being explored on d 14 and 21 of refrigeration. When interpreted together, the indicator organisms and the microbiome have the potential to provide a more thorough description of shelf-life extension through the use of inorganic acids. Although the use of pH, colorimetric, and texture measurements would have provided additional physiochemical variables, these metrics were reserved for future and more expansive studies, where regression analyses could be implemented as a final validation step.

Using the standard indicators of spoilage, total aerobic mesophiles, and LAB, spoilage, as defined by 7 Log_10_ CFU/g of product, occurred between days 7 and 14 (Figures 1, 2). The typical shelf-life of raw bone-in, skin-on broiler wings is between d 7 and 14. Therefore, the occurrence of spoilage (> 7 Log_10_ CFU/g) in the current project is within the projected range of shelf-life. When evaluating total mesophiles alone (Figure 1), the treatment of wings with short-duration (15 s) antimicrobial dips in PAA, SBS, and SBS + PAA resulted in the extended shelf-life of the wings to day 14, while those treated with NT and TW were spoiled by d 7 as indicated by the level of total aerobic mesophiles recovered. When only considering the mitigation of LAB bacteria, the treatment of wings with SBS and SBS + PAA had the most profound effect on the reduction of LAB over the 21-day period (*p* < 0.05; Figure 2).

It is apparent that there are minimal differences in the efficacy of treatments reducing total aerobic mesophiles, but there may be an advantage of including SBS as a short duration dip (15-s) alone or in combination with PAA as it may decrease LAB levels more effectively past d 14 of shelf-life. PAA has previously been demonstrated to extend the shelf-life of broiler carcasses when used during immersion chilling (1 h) at 200 ppm (d 15: 5.89 ± 0.31 Log_10_ CFU/sample) compared with those treated with tap water (d 15: 6.88 ± 0.78 Log_10_ CFU/ml), despite not being significantly different (Oksanen et al., 2018). As no comparison between PAA and SBS on these shelf-life microorganisms of raw poultry products exists beyond the current study, the current research demonstrates a potentially selective efficacy against LAB compared with aerobic mesophiles.

A combinatorial effect of SBS + PAA was evident based on the current data; however, more analyses evaluating the combinatorial effects are required. These results are in congruence with Dittoe et al. (2019a), who determined that the use of 3% SBS, 2% SBS with the addition of 200 ppm PAA, and 3% SBS with the addition of 200 ppm PAA had a significant effect on foodborne pathogens, such as *Salmonella* Enteritidis among raw bone-in poultry drumsticks. Therefore, Dittoe et al. (2019a) summarized that there could be a synergistic effect of adding SBS with PAA. If proven true with more research studies, this combinatorial effect could be due to the independent modes of action having an added benefit effect. Likely, SBS leads to a loss in the ability to maintain cellular osmolarity due to its extremely low pKa of 1.9 (Knueven, 1999), resulting in cytolysis, whereas PAA is primarily thought to work as an oxidizer, resulting in cell death by disrupting cell wall permeability (Middleton et al., 1997; Block, 2001). The use of PAA, an oxidizer, may also result in the denaturation of proteins and oxidation of sulfhydryl and sulfur bonds in proteins, enzymes, and other metabolites (Middleton et al., 1997; Block, 2001).

Additionally, the use of an inorganic acid such as SBS may also be able to counteract the buffering capacity of chicken skin which has the potential to reduce the efficacy of different antimicrobial hurdles (Tan et al., 2014a,b). Thus, the current research employed skin-on poultry parts rather than skinless to demonstrate the protective effect of the skin, allowing bacterial attachment and subsequently protecting those attached cells (Tan et al., 2014a,b). Tan et al. (2014b) demonstrated the use of organic acids, such as acetic acid was capable of reducing *Salmonella* Typhimurium levels better on meat and fat (~1.5 to 7 Log_10_ CFU/g) than that of skin remnants (~1 Log_10_ CFU/g) as the skin remnants had a stronger buffering capacity (13 mmol H^+^/(pH*kg)) than chicken meat and fat (7 mmol H^+^/(pH*kg) and 6.9 mmol H^+^/(pH*kg), respectively). Therefore, in the current study, the use of SBS was demonstrated to be an effective antimicrobial hurdle on skin-on poultry parts in comparison to the industry standard, PAA, with both extending shelf-life 7 days past the NT and TW controls.

### 4.2 Peracetic acid and SBS differentially impact the microbiota’s evenness but not richness

One of the first avenues for understanding microbial ecology is through the evaluation of alpha diversity, which is the evenness and richness of a microbial community. The treatment groups induced significant changes in community richness and evenness but not in time (*p* < 0.05). The richness of the wings treated with NT and TW was higher than that of those treated with PAA and SBS, with the richness not differing between those treated with PAA and SBS (Figure 3A). These results are in congruence with Kim et al. (2017), who determined no difference in richness between broiler carcasses treated post-chill with PAA and Amplon (Zoetis, Parsippany, New Jersey, United States). Amplon is a product similar to SBS in that it is comprised of sulfuric acid and sodium sulfate (4.5–5.5% sodium sulfate, 38.5–39.5% sulfuric acid). Weinroth et al. (2019) determined the use of Amplon, at a pH of 1.2, reduced Faith’s PD, another measure of richness, of ground beef under MAP packaging compared to those treated as the control or with 350 ppm of PAA. Weinroth et al. (2019) attributed these differences to the ability of these different antimicrobials to target different microorganisms by altering the pH of the ground beef. Although pH was not measured in the current study, the buffering capacity of the poultry skin (Weinroth et al., 2019) and the increase in PAA to 500 ppm may have been attributed to the lack of significance between the use of PAA and SBS on the richness of the microbial community. Pielou’s Evenness paralleled the richness findings, with rinsates of wings treated with SBS and SBS + PAA having the least amount of evenness as compared with the other groups (*p* < 0.05; *Q* < 0.05; Figure 3B).

### 4.3 Peracetic acid and SBS differentially impact microbiota diversity over time

Beta diversity indices provide information on the non-descriptive compositional variation of that community structure and variance across a population. Using ADONIS, a multivariate analysis can determine that if main or interactive effects are driving beta diversity metrics, there was an interaction between treatment and time on the beta diversity of the microbial communities, with time being a significant driver for those effects (*p* < 0.05; Supplementary Table S3). As such, volatility plots were created to visualize shifts over time (Figure 4). As expected, the most readily spoiled poultry parts, the control groups, did not change drastically over time (d 14 to 21). However, those treated demonstrated differences over time, with PAA slightly increasing in diversification over time and SBS and SBS + PAA sustaining or reducing the microbial compositional diversity. Although the current study was not intended to compare culture-dependent and culture-independent analyses, the aerobic mesophile and LAB counts correspond well with the stability demonstrated through the volatility plots. As the controls, NT and TW, were on the verge of spoilage on d 7, the d 14 and 21 microbial composition should not have shifted significantly. However, those treated did not reach the end of their shelf-life until d 14 or 21. Therefore, the stability of a population could be indicative of spoilage. Unlike the current study, Weinroth et al. (2019) did not demonstrate differences in the beta diversity, Weighted Unifrac, or ground beef before (15 d post-grind) and after retail display (26 d post-grind). However, Weinroth et al. (2019) demonstrated distinct Weighted Unifrac Distances between ground beef treated as the control, with 350 ppm PAA or with sulfuric acid, and sodium sulfate blend at a pH of 1.2 irrespective of time.

### 4.4 Compositional changes by time support microbiological and diversity results

At the end of shelf-life (d 12 and 16), raw poultry products have been reported to be comprised of *Brochothrix*, *Carnobacterium*, *Vagococcus*, and *Janthinobacterium* when using culture-independent methods, such as microbiome sequencing (Lauritsen et al., 2019). Additionally, at 10 d of shelf-life under aerobic conditions, broiler legs treated with water or PAA (13.67 mM) have been demonstrated to be comprised of *Pseudomonas*, *Psychrobacter*, *Carnobacterium*, *Enterobacteriaceae*, and *Serratia* (Zhang et al., 2022). The reported findings are congruent with the current research, where 74 taxa were identified at the species level, with 4 and 14 differentially abundant taxa at the class and species levels (Figure 6).

In the current study, it was evident that the use of antimicrobials such as PAA or SBS as short-duration dips does alter the microbial composition. As observed with Kim et al. (2017), the current study saw the emergence of taxa belonging to Bacteroides (*Monocercomonoides* sp. *PA203*) and *Bacillus* (*Aneurinibacillus thermophilus*, *Bacillales*, and *Janthinobacterium* spp.), emerging in the PAA and SBS treatment groups. Those treated with SBS and SBS + PAA had a greater abundance of Bacilli, such as *Bacillus* spp., *Aneurinibacillus thermoaerophilus*, and *Bacillales*, than other groups. The addition of PAA had similar relative abundance of species belonging to Gammaproteobacteria as those treated with NT and TW on d 14. Additionally, those treated with PAA had a greater abundance of *Shewanella* spp. and *Acinetobacter* spp. on d 14; however, on d 21, those treated with PAA were predominated by *Enterobacteriaceae* and *Janthinobacterium* spp. Meanwhile, those treated with SBS were more abundant with *Shewanella* spp., and those treated with SBS + PAA were more abundant in *Shewanella* spp. and *Enterobacteriaceae* on d 21. Although the NT and TW groups demonstrated changes in composition, these changes were not as dramatic as the changes were observed in composition among those treated, reinforcing the beta and alpha diversity results observed in the current study.

### 4.5 Inorganic acid, SBS, selectively enriches for bacilli and not lactobacilli

Weinroth et al. (2019) demonstrated that the spray treatment of trim prior to grinding significantly impacted the families such as Enterobacteriaceae, Lactobacillaceae, and Leuconostocaceae with the ground beef treated with Amplon and the blend of sulfuric acid and sodium sulfate, having a higher normalized sequence count of Lactobacillaceae and Leuconostocaceae than those treated as the control or with 350 ppm PAA (ANCOM, *W* = 8). Additionally, those treated with Amplon or PAA had lower Enterobacteriaceae normalized counts than those treated as the control (Weinroth et al., 2019). Weinroth et al. (2019) hypothesized that the use of weak organic acids, such as Amplon, is more effective against Gram-negative microorganisms compared with Gram-positives and, thus, was the reason for the inability of Amplon to reduce these populations. Although Amplon and SBS are similar in chemical composition, the application in the current study did not result in significant differences in these populations. Additionally, Weinroth postulated that the pH of the Amplon treatment (1.2 pH) would have altered the pH of the ground beef, and the LAB populations would be altered. Although the pH of the meat was not measured in the current study, the buffering capacity of chicken skin would likely inhibit the LAB effects, which was demonstrated in ground beef (Tan et al., 2014a,b). The use of SBS and SBS + PAA on chicken wings decreased culture-dependent LAB levels compared with those treated as the controls or treated with PAA. Therefore, the observations made by Weinroth et al. (2019) may not apply to poultry products with beef.

As previously mentioned, the current study did not observe an increase in LAB, according to Weinroth et al. (2019); however, the current study observed an increase in a gram-positive population within the class of Bacilli among wings treated with SBS and SBS + PAA on d 14 and 21. Within the class of Bacilli are several acid-resistant spore-forming spoilage bacteria, such as those within the species of *Bacillus subtilis* (Sun et al., 2021). During poultry processing, spore formers such as *Bacillus* spp. are prevalent among the carcass microbiome (Kim et al., 2017). However, the use of an inorganic acid with an extremely low pKa, such as SBS, may inhibit typical spoilage-associated microorganisms such as *Aeromonas*, *Shewanella*, *Enterobacteriaceae*, *Janthinobacterium*, *Acinetobacter*, *Escherichia-Shigella*, and *Psychrobacter* spp. may not be able to mitigate acid-tolerant spore formers. With no competitors, these acid-tolerant spore-forming spoilage bacteria are capable of sporulating and dominating the microbiome of chicken wings. This phenomenon is becoming more evident with multiple food matrices (André et al., 2017; Sagdic et al., 2017) and has been gathering increased awareness across the food industry (Zhang and Mathys, 2019).

## 5 Conclusion

Spoilage of poultry products has generally been defined by physical characteristics (slime, off-odor, and texture) and microbial abundances (LAB and aerobic mesophiles). All these metrics have proven to be essential components for determining the onset of spoilage and measuring the impact of shelf-life extension efforts. However, as new technologies emerge, the ability to improve the understanding of how shelf-life extension efforts impact the microbial ecology of poultry products and subsequent spoilage has surfaced. By discerning the impact different antimicrobials have on shelf-life in terms of microbial ecology and stability, poultry processing personnel can make more informed decisions at the facility level.

Although the current study is a preliminary evaluation of the antimicrobial effects of SBS and the combination of SBS and PAA on the microbial ecology of poultry products using culture-dependent and independent means, the treatment of bone-in, skin-on chicken wings with 3% SBS increased the shelf-life of these poultry products for 7 days more than those treated with NT and TW by microbiological standards, with spoilage occurring between d 14 and day 21 post-treatment. In fact, the wings treated with SBS and SBS + PAA exhibited LAB levels approximately 3 Log_10_ CFU/g on d 7 and 14. Regardless, by d 21, there was a significant increase in microbial populations among all groups. These changes in traditional microbiological plating were in parallel to the changes identified among the microbiota.

While microbiome sequencing may not be a rapid screen for spoilage, understanding antimicrobial effects on that population and how spoilage may be delayed or impacted is essential for designing a targeted multi-hurdle approach. In the current study, microbiome sequencing revealed the selective enrichment of Bacilli, a class comprised of acid-resistant spore-forming spoilage bacteria, with the use of SBS as a short-duration antimicrobial dip (15 s) on skin-on, bone-in poultry wings while reducing other spoilage-associated microorganisms. Between d 14 and 21 of shelf-life, the microbial ecology of the treated wings was collapsing with both volatility and ANCOM results, demonstrating the increase in spoilage microorganisms among treated wings, whereas the controls maintained static from d 14 to 21 as the shelf-life on untreated wings had already been reached by d 14. Additionally, those treated with SBS alone or in combination with PAA demonstrated a loss of Clostridia and Bacilli selective enrichment and the emergence and predominance of spoilage populations on d 21 of shelf-life.

In conclusion, using SBS as a short-duration (15 s) antimicrobial dip is an effective shelf-life extension tool on bone-in, skin-on chicken wings compared with the industry standard, PAA. More specifically, the use of SBS as an antimicrobial: (1) resulted in the increase in shelf-life by 7 days compared to untreated chicken wings; (2) is more effective in reducing culture-dependent (LAB plate data) and culture-independent (microbiome data) populations compared to the industry standard PAA on d 14 of shelf-life but these effects were gone by the end of shelf-life, d-21; and (3) potentially selects for acid-tolerant spore-forming spoilage bacteria within the class of Bacilli on d 14. Therefore, the use of SBS as a short-duration (15 s) antimicrobial dip on poultry products is an attractive shelf-life extension hurdle that warrants further research and eventual integration into poultry processing facilities.

## Data availability statement

The datasets presented in this study can be found in online repositories. The names of the repository/repositories and accession number(s) can be found at: https://www.ncbi.nlm.nih.gov/, PRJNA847567; https://github.com, https://github.com/RickeLab-UW/Microbiome-of-Treated-Chicken-Wings.

## Ethics statement

Ethical approval was not required for the studies on animals in accordance with the local legislation and institutional requirements because carcass parts were procured from a commercial processor.

## Author contributions

DD: Writing – original draft, Writing – review & editing, Conceptualization, Data curation, Formal analysis, Investigation, Methodology, Project administration, Visualization. KF: Conceptualization, Formal analysis, Project administration, Supervision, Writing – original draft, Writing – review & editing. CO: Conceptualization, Funding acquisition, Resources, Writing – review & editing. HT: Data curation, Project administration, Writing – review & editing. SR: Conceptualization, Funding acquisition, Project administration, Resources, Supervision, Writing – review & editing.

## Footnotes

^1^ https://github.com/RickeLab-UW/Microbiome-of-Treated-Chicken-Wings
