# Supplementary material for: Exploiting the microbiota of organic and inorganic acid-treated raw poultry products to improve shelf-life
Source: Front Microbiol. 2024 Feb 27;15:1348159. doi: 10.3389/fmicb.2024.1348159 (PMC10927844; doi:10.3389/fmicb.2024.1348159)
Supplement: Supplementary file 1 [file Data_Sheet_1.docx]

Supplementary Material

# Supplemental Figures

Supplemental Figure 1. Rarefaction plot used to determine where to determine max depth for alpha and beta diversity core metrics performed in QIIME2. Rarefaction depth was set to the average sequencing depth, 100,000, and the max depth for the core metrics was set at 200 to retain all samples as described in the methodology.

# Supplemental Tables

Supplemental Table 1. Interaction of treatment and time on the shelf-life (aerobic mesophiles and lactic acid bacteria) of poultry wings treated with organic (PAA) and inorganic acids (SBS) and their combination (SBS + PAA).

|  | | Aerobic Mesophiles | | Lactic Acid Bacteria | | |
| --- | --- | --- | --- | --- | --- | --- |
| Day | Treatment | Mean | ± SEM | Mean | ± SEM | |
| 0 | NT | 4.22 | ± 0.05^de^ | 3.10 | ± 0.06^g^ |  |
|  | TW | 4.22 | ± 0.04^de^ | 3.21 | ± 0.10^g^ |  |
|  | SBS | 4.26 | ± 0.04^de^ | 3.07 | ± 0.05^g^ |  |
|  | PAA | 4.05 | ± 0.03^d^ | 3.12 | ± 0.07^g^ |  |
|  | SBS + PAA | 4.13 | ± 0.04^de^ | 3.19 | ± 0.05^g^ |  |
| 7 | NT | 6.66 | ± 0.12^b^ | 5.02 | ± 0.08^e^ |  |
|  | TW | 6.72 | ± 0.09^b^ | 4.79 | ± 0.22^ef^ |  |
|  | SBS | 4.10 | ± 0.30^c^ | 2.96 | ± 0.03^g^ |  |
|  | PAA | 5.18 | ± 0.23^de^ | 3.01 | ± 0.05^g^ |  |
|  | SBS + PAA | 3.54 | ± 0.07^e^ | 2.99 | ± 0.03^g^ |  |
| 14 | NT | 7.81 | ± 0.09^a^ | 6.17 | ± 0.18^cd^ |  |
|  | TW | 7.98 | ± 0.09^a^ | 6.50 | ± 0.20^bc^ |  |
|  | SBS | 5.29 | ± 0.18^b^ | 3.03 | ± 0.05^f^ |  |
|  | PAA | 6.48 | ± 0.15^c^ | 4.20 | ± 0.28^g^ |  |
|  | SBS + PAA | 6.98 | ± 0.16^b^ | 3.12 | ± 0.14^g^ |  |
| 21 | NT | 8.33 | ± 0.05^a^ | 7.19 | ± 0.11^ab^ |  |
|  | TW | 8.34 | ± 0.05^a^ | 7.55 | ± 0.25^a^ |  |
|  | SBS | 8.10 | ± 0.11^a^ | 6.48 | ± 0.22^de^ |  |
|  | PAA | 7.80 | ± 0.13^s^ | 5.39 | ± 0.18^bc^ |  |
|  | SBS + PAA | 7.75 | ± 0.13^a^ | 5.05 | ± 0.09^e^ |  |

Supplemental Table 2. Main effect and interaction of treatment and day on the richness and evenness.

|  |  | Shannon’s Entropy | | | Pielou’s Evenness | | |
| --- | --- | --- | --- | --- | --- | --- | --- |
|  | DF^1^ | SS | F-value | P-value | SS^2^ | F-value | P-value |
| Treatment | 4 | 10.67 | 12.45 | < 0.001 | 390.68 | 3.36 | 0.02 |
| Day | 1 | 0.003 | 0.01 | 0.91 | 35.28 | 1.22 | 0.28 |
| Treatment × Day | 4 | 0.85 | 0.99 | 0.43 | 60.92 | 0.52 | 0.72 |
| Residual | 40 | 8.57 |  |  | 1162.8 |  |  |

^1^Degrees of freedom

^2^Sums of Squares

Supplemental Table 3. Pairwise differences between the richness and evenness of bone-in, skin-on chicken wings untreated or treated with organic and inorganic acids.

|  |  | Shannon's Diversity | | | Pielou's Evenness | | |
| --- | --- | --- | --- | --- | --- | --- | --- |
| Group 1 | Group 2 | H^2^ | P-value | Q-value | H | P-value | Q-value |
| NT (n=10) | PAA (n=10) | 13.720 | **< 0.001** | **0.001** | 4.166 | **0.041** | 0.069 |
|  | SBS (n=10) | 13.720 | **< 0.001** | **0.001** | 11.571 | **0.001** | **0.006** |
|  | SBS+PAA (n=10) | 4.806 | **0.028** | 0.057 | 8.251 | **0.004** | **0.014** |
|  | TW (n=10) | 1.120 | 0.290 | 0.322 | 0.006 | 0.940 | 0.940 |
| PAA (n=10) | SBS (n=10) | 3.291 | 0.070 | 0.087 | 7.000 | **0.008** | **0.020** |
|  | SBS+PAA (n=10) | 0.051 | 0.821 | 0.821 | 1.286 | 0.257 | 0.285 |
|  | TW (n=10) | 12.623 | **< 0.001** | **0.001** | 3.023 | 0.082 | 0.103 |
| SBS (n=10) | SBS+PAA (n=10) | 4.166 | **0.041** | 0.059 | 3.023 | 0.082 | 0.103 |
|  | TW (n=10) | 12.623 | **< 0.001** | **0.001** | 10.566 | **0.001** | **0.006** |
| SBS+PAA (n=10) | TW (n=10) | 4.166 | **0.041** | 0.059 | 5.851 | **0.016** | **0.031** |

^1^Bolded values are those with significant pairwise differences (P- and Q-values)

^2^H value is the test statistic for the Kruskal Wallis test.

Supplemental Table 4. Main effect and interaction of treatment and day on the beta diversity of bone-in, skin-on chicken wings when using ADONIS .

|  |  | Bray Curtis | | | | | Jaccard | | | | | Unweighted Unifrac | | | | | Weighted Unifrac | | | | |
| --- | --- | --- | --- | --- | --- | --- | --- | --- | --- | --- | --- | --- | --- | --- | --- | --- | --- | --- | --- | --- | --- |
|  | Df^1^ | SS^2^ | MS^3^ | F-Model | R^2^ | P-value^4^ | SS | MS | F-Model | R^2^ | P-value | SS | MS | F-Model | R^2^ | P-value | SS | MS | F-Model | R^2^ | P-value |
| Treatment | 4 | 1.321 | 0.330 | 5.713 | 0.316 | **0.001** | 2.884 | 0.721 | 3.761 | 0.234 | **0.001** | 2.140 | 0.535 | 3.886 | 0.236 | **0.001** | 0.027 | 0.027 | 4.335 | 0.058 | **0.006** |
| Day | 1 | 0.151 | 0.151 | 2.604 | 0.036 | **0.036** | 0.437 | 0.437 | 2.279 | 0.035 | **0.015** | 0.484 | 0.484 | 3.515 | 0.053 | **0.003** | 0.152 | 0.038 | 6.044 | 0.326 | **0.001** |
| Treatment × Day | 4 | 0.394 | 0.099 | 1.705 | 0.094 | **0.045** | 1.342 | 0.335 | 1.750 | 0.109 | **0.004** | 0.932 | 0.233 | 1.692 | 0.103 | **0.025** | 0.036 | 0.009 | 1.411 | 0.076 | 0.154 |
| Residuals | 40 | 2.313 | 0.058 |  | 0.553 |  | 7.667 | 0.192 |  | 0.622 |  | 5.507 | 0.138 |  | 0.608 |  | 0.252 | 0.006 |  | 0.539 |  |
| Total | 49 | 4.179 |  |  | 1.000 |  | 12.329 |  |  | 1.000 |  | 9.063 |  |  | 1.000 |  | 0.467 |  |  | 1.000 |  |

^1^Degrees of freedom

^2^Sums of Squares

^3^Mean Squares

^4^Bolded values are those with significant P-values.

Supplemental Table 5. Pairwise differences between the beta diversity of bone-in, skin-on chicken wings untreated or treated with organic and inorganic acids.

|  |  |  |  | Bray Curtis | | | Jaccard Dissimilarity | | | Unweighted Unifrac | | | Weighted Unifrac | | |
| --- | --- | --- | --- | --- | --- | --- | --- | --- | --- | --- | --- | --- | --- | --- | --- |
| Group 1 | Group 2 | N | Permutations | R^1^ | P-value^2^ | Q-value^2^ | R | P-value | Q-value | R | P-value | Q-value | R | P-value | Q-value |
| NT | PAA | 20 | 999 | 0.373 | **0.001** | **0.002** | 0.674 | **0.001** | **0.003** | 0.405 | **0.001** | **0.003** | 0.592 | **0.001** | **0.003** |
|  | SBS | 20 | 999 | 0.769 | **0.001** | **0.002** | 0.672 | **0.001** | **0.003** | 0.546 | **0.001** | **0.003** | 0.564 | **0.001** | **0.003** |
|  | SBS + PAA | 20 | 999 | 0.267 | **0.001** | **0.002** | 0.270 | **0.003** | **0.005** | 0.135 | **0.012** | **0.020** | 0.232 | **0.002** | **0.004** |
|  | TW | 20 | 999 | -0.003 | 0.466 | 0.466 | -0.047 | 0.779 | 0.779 | -0.040 | 0.801 | 0.801 | -0.031 | 0.635 | 0.635 |
| PAA | SBS | 20 | 999 | 0.346 | **0.003** | **0.004** | 0.137 | **0.019** | **0.027** | -0.008 | 0.480 | 0.533 | 0.051 | 0.084 | 0.105 |
|  | SBS + PAA | 20 | 999 | 0.118 | **0.035** | **0.039** | 0.064 | 0.126 | 0.158 | 0.051 | 0.193 | 0.241 | 0.108 | **0.016** | **0.023** |
|  | TW | 20 | 999 | 0.440 | **0.001** | **0.002** | 0.629 | **0.001** | **0.003** | 0.316 | **0.004** | **0.010** | 0.665 | **0.001** | **0.003** |
| SBS | SBS + PAA | 20 | 999 | 0.125 | **0.032** | **0.039** | 0.006 | 0.361 | 0.401 | 0.094 | 0.097 | 0.139 | 0.019 | 0.266 | 0.296 |
|  | TW | 20 | 999 | 0.749 | **0.001** | **0.002** | 0.657 | **0.001** | **0.003** | 0.481 | **0.001** | **0.003** | 0.632 | **0.001** | **0.003** |
| SBS + PAA | TW | 20 | 999 | 0.282 | **0.002** | **0.003** | 0.267 | **0.002** | **0.004** | 0.131 | **0.012** | **0.020** | 0.269 | **0.004** | **0.007** |

^1^R is the ANOSIM statistic, which compares the mean of ranked dissimilarities between groups to the mean of ranked dissimilarities within groups.

^2^Bolded values are those with significant P and Q values.
